# GLP-1/glucagon receptor co-agonism for treatment of obesity

**DOI:** 10.1007/s00125-017-4354-8

**Published:** 2017-07-21

**Authors:** Miguel A. Sánchez-Garrido, Sara J. Brandt, Christoffer Clemmensen, Timo D. Müller, Richard D. DiMarchi, Matthias H. Tschöp

**Affiliations:** 1 Institute for Diabetes and Obesity, Helmholtz Diabetes Center at Helmholtz Zentrum München, German Research Center for Environmental Health (GmbH), Business Campus Garching, Parkring 13, 85748 Garching, Germany; 2 German Center for Diabetes Research (DZD), Neuherberg, Germany; 3 Department of Chemistry, Indiana University, 800 E Kirkwood Ave, Bloomington, IN 47405 USA; 4 Division of Metabolic Diseases, Department of Medicine, Technische Universität München, Munich, Germany

**Keywords:** Co-agonism, Dual agonism, GLP-1, Glucagon, Multi-agonist, Obesity, Pharmacology, Review, Translational, Type 2 diabetes

## Abstract

Over a relatively short period, obesity and type 2 diabetes have come to represent a large medical and economic burden to global societies. The epidemic rise in the prevalence of obesity has metabolic consequences and is paralleled by an increased occurrence of other diseases, such as diabetes, cancer and cardiovascular complications. Together, obesity and type 2 diabetes constitute one of the more preventable causes of premature death and the identification of novel, safe and effective anti-obesity drugs is of utmost importance. Pharmacological attempts to treat obesity have had limited success, with notable adverse effects, rendering bariatric surgery as the only current therapy for substantially improving body weight. Novel unimolecular, multifunctional peptides have emerged as one of the most promising medicinal approaches to enhance metabolic efficacy and restore normal body weight. In this review, we will mainly focus on the discovery and translational relevance of dual agonists that pharmacologically function at the receptors for glucagon and glucagon-like peptide-1. Such peptides have advanced to clinical evaluation and inspired the pursuit of multiple related approaches to achieving polypharmacy within single molecules.

**Electronic supplementary material:**

The online version of this article (doi:10.1007/s00125-017-4354-8) contains a slide of the figure for download, which is available to authorised users.

## Introduction

Obesity and diabetes represent dire threats to public health. In 2014, the WHO estimated that there were 1.9 billion overweight and 600 million obese people worldwide [1], representing 39% of the global population [1]. Obesity has direct links to hypertension, cardiovascular disease, certain types of cancers and, most predominantly, type 2 diabetes [2, 3]. In 2014, there were 422 million people with diabetes (8.5% of the global population) [4] and this number is expected to rise to 592 million by 2035 [5].

While often concurrent in a single person, each of these diseases is individually managed and this is likely to continue until the intransigence of obesity is successfully addressed. Once a person is overweight or, worse, obese, it is extremely difficult to permanently reverse the weight gain. Lifestyle interventions, anchored on diet and exercise, typically provide a small, short-lasting weight loss [6]. At the other extreme, bariatric surgeries are highly effective in reducing body weight and improving glucose tolerance in most individuals [7, 8]. However, such intervention is highly invasive, has considerable risk and is irreversible and expensive. Therefore, surgery is recommended only for individuals who are severely obese (BMI > 40 kg/m^2^) or who have a BMI of 35–40 kg/m^2^ together with severe comorbidities such as cardiopulmonary disease or diabetes [9].

For people who do not meet the eligibility criteria for bariatric surgery and who fail to maintain weight loss through lifestyle interventions, pharmacotherapy is the only remaining option. Current federally approved pharmacotherapies are reported to result in 5–15% body weight loss [10]. Anti-obesity and glucose-lowering pharmacotherapies predominantly exert their actions by inducing satiety (liraglutide, setmelanotide, lorcaserin, pramlintide, sibutramine) or by inducing malabsorption of nutrients (orlistat). When sustained, this degree of weight loss can provide meaningful improvements in metabolism and lipid management [11]. However, these therapies are commonly accompanied by adverse gastrointestinal and cardiovascular effects that limit their use [12]. The ultimate goal is to identify medicinal therapy that approaches the effectiveness of bariatric surgery without its associated complexities, adverse effects or financial burden.

It is logical that improved efficacy can be achieved by combining multiple metabolic actions within a single therapy; indeed, there is precedent for this [13]. The challenge has been to minimise and balance the pharmacology to select a combination that maximises the benefit without risk of irreversible toxicity, as historically experienced in co-therapy with fenfluramine and phentermine. In this review, we discuss the discovery and development of the first rationally designed approach to unimolecular co-agonism, which recruits the well-established pharmacology of glucagon-like peptide 1 (GLP-1) and, counterintuitively, glucagon.

## Physiology, pharmacology and clinical relevance of GLP-1

GLP-1 is a peptide hormone produced within the L cells of the intestine. GLP-1 is derived from proglucagon, and proteolytic processing results in the biologically active form, consisting of 30 amino acids. There is some ambiguity in the literature when numbering the amino acids within GLP-1, as the prohormone is synthesised with 37 amino acids. The endogenous cleavage of the first six residues results in the biologically active peptide, which represents GLP-1 (7–36) amide. Consequently, two numbering systems have been used where the seventh amino in the precursor and the first in active hormone are the same. In this text, we use the numbering whereby the amino acids that constitute active GLP-1 represent positions 1–30 and are the same as 7–36 in the precursor.

GLP-1 is secreted in response to nutrient ingestion, especially in response to meals high in fat and carbohydrates [14]. GLP-1 functions to delay gastric emptying, stimulate insulin secretion and mediate satiety in the central nervous system (CNS), all actions that are beneficial to individuals with obesity and type 2 diabetes. In type 2 diabetes, preprandial administration of native GLP-1 reduces plasma glucose and improves glucose tolerance [15]. However, due to its short circulating half-life, native GLP-1 is ill-suited for chronic therapy [14]. The short half-life (1–2 min in humans) results from dipeptidylpeptidase-IV (DPP-IV) proteolysis by which the N-terminal dipeptide is rapidly removed, inactivating GLP-1. GLP-1 is also cleared relatively quickly by renal filtration [16]. To effectively use GLP-1 as a drug, several modifications have been made to address proteolysis and clearance. Substitution with the non-native amino acid aminoisobutyric acid at position 2 confers resistance to DPP-IV degradation [17]. Other chemical modifications include increasing the size of the hormone or promoting non-covalent attachment to serum albumin, thus extending the plasma circulation time [18, 19].

An alternative approach to enable oral administration employs DPP-IV inhibitors to preserve endogenous GLP-1. Currently, there are several registered DPP-IV inhibitors, including sitagliptin, saxagliptin and linagliptin [20]. Studies comparing DPP-IV inhibitors with GLP-1 receptor (GLP-1R) agonists have shown greater weight loss and reduction in glucose excursions in individuals treated with GLP-1R agonists [20]. This reflects the ability to more intensively supplement physiological levels with GLP-1R agonists, rendering them the more powerful approach for treating obesity and type 2 diabetes. There are six GLP-1-based medicines currently approved by the US Food and Drug Administration (FDA): exenatide, lixisenatide, liraglutide, semaglutide, dulaglutide and albiglutide (Table 1). Each of these has been optimised by a specific method to extend the duration of action of GLP-1 following a single injection.

**Table 1:** GLP-1-based mono-agonists and poly-agonists in preclinical development or used clinically for obesity and type 2 diabetes therapy

Drug	Company	Target	Phase	Administration
Exenatide (Byetta)	AstraZeneca	GLP-1R	Registered	SC, twice daily
Exenatide (Bydureon)	AstraZeneca	GLP-1R	Registered	SC, weekly
Lixisenatide (Lyxumia)	Sanofi-Aventis	GLP-1R	Registered	SC, daily
Liraglutide (Victoza)	Novo Nordisk	GLP-1R	Registered	SC, daily
Semaglutide	Novo Nordisk	GLP-1R	Phase 3	Oral, daily
Semaglutide	Novo Nordisk	GLP-1R	Phase 3	SC, weekly
Dulaglutide (Trulicity)	Eli Lilly	GLP-1R	Registered	SC, weekly
Albiglutide (Tanzeum)^a^	GlaxoSmithKline	GLP-1R	Registered	SC, weekly
SAR425899	Sanofi-Aventis	GLP-1R/GcgR	Phase 1	SC, daily
LY2944876/TT-401	Eli Lilly	GLP-1R/GcgR	Phase 2	SC, weekly
HM12525A	Hanmi Pharmaceuticals	GLP-1R/GcgR	Phase 1	SC, weekly
ZP2929	Zealand	GLP-1R/GcgR	Phase 1	SC, daily
MEDI0382	MedImmune	GLP-1R/GcgR	Phase 1	SC
VPD-107	Spitfire Pharma	GLP-1R/GcgR	Preclinical	SC, weekly
MOD-6031	OPKO Biologics	GLP-1R/GcgR	Phase 1	SC, monthly
Liraglutide + NN9030	Novo Nordisk	GLP-1R + GcgR	Phase 1	SC
Cpd86	Eli Lilly	GLP-1R/GIPR	Preclinical	SC
ZP-DI-70	Zealand	GLP-1R/GIPR	Preclinical	SC, weekly
NN9709/MAR709	Novo Nordisk/Marcadia	GLP-1R/GIPR	Phase 2	SC, daily
MAR423	Novo Nordisk/Marcadia	GLP-1R/GIPR/GcgR	Phase 1	SC, daily

^a^Also available as Eperzan

GIPR, Glucose-dependent insulinotropic polypeptide receptor; SC, subcutaneous

Exenatide (Byetta, AstraZeneca) is a synthetic version of exendin-4, a GLP-1R agonist found in the saliva of the Gila monster (*Heloderma suspectum*). Exenatide contains a glycine at position 2, rendering it less susceptible to DPP-IV degradation, and includes a C-terminal extension (CEX), which stabilises the secondary structure of the hormone, increasing its solubility in physiological buffer. These modifications extend the half-life of the hormone to 2.4 h [18]. Exenatide is also available as microsphere depot formulation (Bydureon, AstraZeneca), which sustains the duration of action suitable for once-weekly administration [21]. Further modifications to exenatide yielded lixisenatide (Lyxumia, Sanofi-Aventis), which has an extended C-terminus including six consecutive lysine residues. Lixisenatide is potent at the GLP-1R and has a half-life of 3–4 h [22]. In clinical trials, both exenatide and lixisenatide reduced body weight by 2.0–3.8 kg and reduced HbA_1c_ levels by 0.5–1.5% (5.5–1.6 mmol/mol) [23–25]. Exenatide was approved by the US FDA in 2005 and by the European Medicines Agency (EMA) in 2006.

Unlike exenatide and lixisenatide, liraglutide (Victoza, Novo Nordisk) maintains enhanced sequence similarity to native human GLP-1. Its main modification is a C16 palmitoyl moiety covalently attached to the lysine at position 20 through a γ-glutamic acid chemical spacer. Liraglutide also includes an arginine substitution at position 28. Since liraglutide retains the native alanine at position 2, it is not directly protected from enzymatic degradation, although palmitoylation and subsequent association with serum albumin results in steric protection [26]. Liraglutide was approved by the EMA in 2009 and the US FDA in 2010 as a glucose-lowering therapy and at a higher dose by the US FDA for the treatment of obesity. In individuals with type 2 diabetes, liraglutide treatment results in a weight loss of 1.3–8.6 kg and a 0.9–2.2% (9.9–24.2 mmol/mol) reduction in HbA_1c_, relative to baseline [27].

Similar to liraglutide, semaglutide (Novo Nordisk) is a fatty-acylated GLP-1 analogue with a stearic-diacid at lysine position 20, linked via a di-aminoethoxy, γ-glutamic acid spacer, and an aminoisobutyric residue at position 2 to provide protection from DPP-IV degradation [28]. Semaglutide has a half-life of 165 h in humans, due in part to non-covalent association with human serum albumin [28]. Preclinical safety studies demonstrated no indication of pancreatitis or pancreatic inflammation in cynomolgus monkeys [29]. Semaglutide has advanced to registration phase clinical trials. In patients with type 2 diabetes, semaglutide is reported to improve glycaemic control dose-dependently, decreasing HbA_1c_ levels by 1.7% (18.7 mmol/mol) vs 0.5% (5.5 mmol/mol) with placebo and 4.8 kg of weight loss vs 1.2 kg with placebo, relative to baseline. The magnitude of these effects was greater than those with open-label liraglutide in the same study [30]. In addition, in a cardiovascular outcome study involving patients with type 2 diabetes, of whom more than 80% had a history of cardiovascular disease, semaglutide treatment lowered the rates of 3-point MACE (myocardial infarction, stroke and cardiovascular death) [31]. Semaglutide has been developed as a once-a-week subcutaneous injection and is currently also being developed for daily oral administration.

Dulaglutide (Trulicity, Eli Lilly) consists of two fused GLP-1 analogues, with a glycine substitution at position 2, a glutamic acid substitution at position 20 and a glycine at position 30, which protect against DPP-IV degradation [19, 32]. The half-life of dulaglutide is greatly extended by its conjugation to the Fc fragment of human IgG4 [18]. This antibody conjugation increases the molecular weight of the molecule well past the renal threshold, providing a half-life of 96 h [18]. In clinical trials, dulaglutide produced a 2.3–3.0 kg body weight loss and a 0.78–1.64% (8.6–18.0 mmol/mol) reduction in HbA_1c_, relative to baseline [33]. Dulaglutide was approved by the US FDA and the EMA in 2014.

Albiglutide (Tanzeum [also available as Eperzan], GlaxoSmithKline) is a fusion of two repeats of GLP-1 and rDNA-derived human albumin. Within the GLP-1 sequences, the alanine at position 2 is substituted with a glycine residue to confer protection against DPP-IV. The half-life of this molecule is 5 days [18]. Studies in individuals with type 2 diabetes revealed a 1.1–1.7 kg reduction in body weight and a 0.79–0.89% (8.7–9.78 mmol/mol) decrease in HbA_1c_ [34]. Albiglutide was approved by the US FDA and the EMA in 2014.

While all of these GLP-1-based medicines demonstrate some success in lowering body weight and HbA_1c_ levels, GLP-1 and its analogues are not without unwanted side effects. The most common adverse effect is nausea. While often mild, the nausea is dose dependent and limits the use of higher doses to drive greater weight loss [35]. GLP-1 therapies may also increase the risk of pancreatitis, although this concern seems to have abated in the last 2 years [36–39]. In this regard, clinical study of lixisenatide in individuals with type 2 diabetes and acute coronary syndrome did not reveal an association with pancreatitis [40]. Similarly, recent long-term studies of liraglutide and semaglutide, focusing on cardiovascular outcomes in individuals with type 2 diabetes, reveal a similar incidence of pancreatitis between groups treated with the GLP-1R agonists and placebo [31, 41].

## Physiology, pharmacology and clinical relevance of glucagon

Glucagon is a peptide hormone secreted by the alpha cells of the pancreas in response to fasting or hypoglycaemia [42]. Its primary physiological role is to raise blood glucose levels by inhibiting insulin secretion and by stimulating hepatic glucose production. Glucagon secretion is reciprocally inhibited by insulin action. Therefore, when insulin resistance occurs (as in obesity and type 2 diabetes) or there is complete absence of endogenous insulin (as in type 1 diabetes) there is relative hyperglucagonaemia. This hyperglucagonaemia is observed in most forms of diabetes, in humans and animal models [43–45].

Glucagon is currently available as an injectable treatment for hypoglycaemia, although it is not widely used given its complex method of administration. Glucagon in powder form must be solubilised immediately before injection, a cumbersome process made more difficult by the symptoms of hypoglycaemia, which include mental confusion or even unconsciousness [46]. Recent advances have altered the solubility of glucagon by substituting more hydrophilic residues into the sequence or by use of the CEX [47]. A more stable, physiologically buffered glucagon solution is additionally attractive for potential use in a bi-hormonal pump for treatment of type 1 diabetes.

Owing to glucagon’s hyperglycaemic and insulin-suppressing effects, the glucagon receptor (GcgR) has historically been a prime target for pharmacological suppression rather than activation. Genetically engineered mice without a functional GcgR exhibit lowered blood glucose levels and severe fasting-induced hypoglycaemia [48]. Furthermore, mice made diabetic by streptozotocin (STZ) administration, but genetically deficient for the GcgR, display normal glucose tolerance [49]. Small-molecule antagonists of the GcgR have reduced glycogen breakdown in vitro [50], and the use of antagonistic antisense oligonucleotide directed to the GcgR in *ob*/*ob* and *db*/*db* mice resulted in reduced blood glucose [44, 51]. Similarly, GcgR antagonists have been reported to reduce blood glucose in STZ-induced diabetic rats [52]. In metabolically healthy men, an infusion of Bay 27-9955, one of the first small-molecule GcgR antagonists, lowered blood glucose in response to a glucagon challenge [53]. Interestingly, recent clinical studies have confirmed the glucose-lowering effects of GcgR antagonism in individuals with type 2 diabetes [54, 55]. However, uncertainties persist regarding the prospects for adverse liver effects that might be inherent to the mechanism of action, as stabilisation of hepatic steatosis would be unwelcome.

In addition to effects on glucose homeostasis, glucagon has both catabolic and thermogenic actions. In humans, intravenous administration of glucagon decreases plasma lipids, cholesterol and arachidonic acid through altered metabolic partitioning [56]. Glucagon administration also decreases hepatic triacylglycerol synthesis in rats [56] and stimulates hormone-sensitive lipase in human and rat white adipocytes to promote lipolysis and the release of NEFA [57, 58]. These fatty acids freely circulate and can be accessed by heart, skeletal muscle, kidneys and liver [56]. The kidneys and liver metabolise the fatty acids, producing ketone bodies as common metabolites [56]. These biological actions define the counter-balancing catabolic role that glucagon serves relative to insulin’s anabolic action.

Glucagon also stimulates energy expenditure. In both rats and humans, infusion of glucagon results in increased oxygen consumption [59, 60]. In vitro studies suggest that this effect is mediated by brown adipose tissue (BAT) [61]. It has also been shown that cold exposure increases plasma glucagon levels, suggesting a role for glucagon in non-shivering thermogenesis [62]. Supporting the role of glucagon in increasing BAT thermogenesis, it has been shown that glucagon administration enhances BAT temperature [63]. However, recent evidence that glucagon increases energy expenditure independently of BAT activation in humans [64] indicates that alternative mechanisms such as futile substrate cycling [65] may underlie glucagon’s thermogenic properties.

In isolation, the catabolic and thermogenic actions of glucagon would be beneficial to individuals who are obese or have type 2 diabetes but these actions are inherently paired with the undesirable stimulation of gluconeogenesis and glycogenolysis. Considering the beneficial effects of GcgR antagonists on glycaemia [66–68], it would seem counterintuitive to employ agonism in a therapy for obesity and, certainly, diabetes. Therefore, to safely harness the attractive catabolic and thermogenic effects of glucagon for treating metabolic disease, a counter-balancing therapy that selectively opposes the risk for glucagon-induced hyperglycaemia is required. In the first undertaking toward that goal, GLP-1 was explored as an ideal pharmacological partner, leading to the purposeful discovery of the first GLP-1R/GcgR co-agonists.

## Co-targeting the GLP-1R and GcgR for obesity treatment

### Unimolecular GLP-1R/GcgR co-agonists for obesity treatment

The search for single molecules and, with recently developed high-tech approaches, single mechanisms for treatment of obesity is ongoing. Unfortunately treatment of complex chronic diseases such as obesity has often proven recalcitrant to attempts to attain the desired health benefits [69]. Combination treatments have become common practice in the treatment of type 2 diabetes, hypertension and other diseases associated with advanced age. It is anticipated that obesity will prove no different, with simultaneous activation of anorectic and thermogenic pathways producing meaningful and sustained clinical outcomes. Mixtures of individual medicines complicate drug development and the magnitude of the problem is virtually overwhelming when there are more than two active entities. A promising pharmacological strategy to circumvent this has been made in the treatment of obesity and the metabolic syndrome, via the integration of multiple mechanisms within a single molecule [18, 69]. A unimolecular approach can provide additional benefits relative to co-administration of individual medicines as there is a single pharmacokinetic profile and metabolic fate and the potential for multiple actions at a single cellular target is more likely. Finally, a major objective along with efficacy is safety and, when compared with separate drugs functioning through a single mechanism, there is the promise of reduced toxicity through a less aggressive effect associated with any one single mechanism of action. Recently developed unimolecular GLP-1R/GcgR co-agonists have superior preclinical efficacy to currently prescribed monotherapies in the treatment of obesity.

### GLP-1 and glucagon promote weight loss through distinct mechanisms of action

GLP-1 is believed to act predominantly in the hypothalamus to induce satiety, whereas the weight-lowering effects of glucagon are mostly driven by its lipolytic and thermogenic effects in liver and the adipose tissue [56]. Notably, these peptides have conflicting effects on glucose homeostasis. While GLP-1 lowers blood glucose levels by promoting insulinotropic actions [70], glucagon promotes hyperglycaemia by stimulating hepatic glucose production [56]. It was hypothesised that integration of these opposing actions into a single molecule might minimise the inherent diabetogenic risk of GcgR agonism. Importantly, GLP-1 and glucagon act through structurally related receptors and both peptides exhibit similar amino acid N-terminal sequences, a domain essential for proper interaction with their receptors. Taken together, the metabolic actions of GLP-1 and glucagon, as well as their structural similarities, inspired the search for single-molecule GLP-1R/GcgR co-agonists as potential candidates for obesity treatment.

A variety of unimolecular GLP-1R/GcgR dual agonists were built using the glucagon amino acid sequence as a template. These glucagon-based chimeric peptides were generated by amino acid substitutions to the glucagon sequence aimed at increasing potency at the GLP-1R and conferring balanced co-agonism at both receptors. Additionally, a 40 kDa polyethylene glycol (PEG) polymer was incorporated at position 24 to extend duration of action and reduce frequency of administration. Amino acid substitution at position 2 eliminated DPP-IV proteolysis [17]. Preclinical studies in diet-induced obese (DIO) mice showed that once-weekly administration of the PEGylated GLP-1R/GcgR co-agonist for 1 month dramatically lowered body weight, compared with vehicle treatment [17]. More importantly, the magnitude of improvement in body weight resulted from increased energy expenditure and decreased food intake, which led to a remarkable loss of fat mass. Furthermore, chronic intervention with the dual-action peptide improved lipid metabolism and hepatic steatosis when compared with a chemically matched peptide having high selectivity for the GLP-1R. Contrary to expectations, glycaemic control was also improved after chronic treatment with the GLP-1R/GcgR co-agonist, which implies that GLP-1 activity protected against glucagon-induced hyperglycaemia [17]. Complementary pharmacological studies in mice lacking the GLP-1R corroborated the essential role of GLP-1R agonism in improving glycaemic control and confirmed that the metabolic improvement associated with co-agonist treatment was due to concerted activation of both receptors [17].

The promise of GLP-1R/GcgR co-agonism in the treatment of obesity was highlighted in studies exploring its effect in concert with leptin therapy. Leptin, an adipocyte-derived hormone known to regulate energy homeostasis, acts in the brain to control energy and glucose metabolism [71]. The discovery of leptin generated great hopes for an effective anti-obesity drug. However, it was found that obese people are hyperleptinaemic, develop central leptin resistance and are unresponsive to leptin therapy [72]. It has recently been shown that a PEGylated GLP-1R/GcgR co-agonist could restore leptin sensitivity in DIO mice chronically maintained on a high-fat diet [73], and this may constitute one of the inherent weight-lowering actions of the co-agonist. Regarding the mechanism of action of the dual agonist, it is well-appreciated that GLP-1 acts centrally and in peripheral tissues to regulate energy and glucose homeostasis [74]. The appetite-suppressing effect of GLP-1 is mediated by its action in the brain, whereas its glucose-lowering effect is mainly attributed to enhancement of glucose-stimulated insulin secretion [74]. In mice lacking the GLP-1R in the CNS, GLP-1R agonism does not lower body weight but its glycaemic benefits are preserved [75]. Therefore, the glycaemic improvement in animals treated with a GLP-1R/GcgR co-agonist is attributable to body-weight-independent and -dependent effects.

**Fig. 1:**
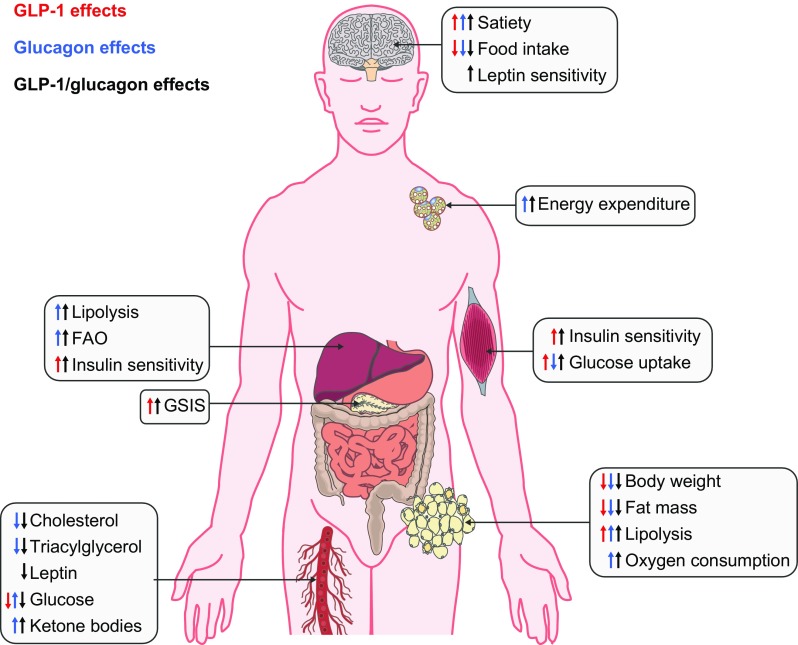
Metabolic actions of GLP-1R agonists and GcgR agonists on key organs (brain, BAT, adipose tissue, muscle, liver, pancreas and circulation) regulating energy and glucose homeostasis and changes in metabolic variables. FAO, fatty acid oxidation; GSIS, glucose-stimulated insulin secretion

Like GLP-1, glucagon acts centrally and peripherally to regulate energy and glucose homeostasis. Glucagon suppresses food intake by acting in the brain [76] and increases energy expenditure by stimulating thermogenesis in peripheral tissues [74]. Hence, the action of dual GLP-1R/GcgR agonists likely results from a combination of central and peripheral mechanisms, at multiple target tissues. The major metabolic actions of dual GLP-1R/GcgR agonists are summarised in Fig. 1. Notably, the metabolic benefits of dual GLP-1R/GcgR agonists have also been documented in obese non-human primates, where chronic administration of a lower dose of a GLP-1R/GcgR co-agonist vs a GLP-1R mono-agonist reduced body weight and improved glucose tolerance to a greater degree [77]. Additional studies in cynomolgus monkeys have confirmed the potent dose-dependent, weight-lowering effects of chronic GLP-1R/GcgR co-agonism [78], supporting the translational promise of this novel balanced agonism for obesity and type 2 diabetes.

Preclinical trials demonstrate that this co-agonist improves glucose tolerance, indicating that the GLP-1 activity can successfully buffer the hyperglycaemic potential of GcgR agonism. Nevertheless, these co-agonists must be carefully monitored as a loss in GLP-1R activity could lead to excessive glucagon-mediated deleterious effects on glucose control. It is especially important to confirm that this therapy is safe and effective in conditions of insulin insensitivity and impaired insulin secretion. While the long-term consequences of GLP-1R agonism are now emerging, similar studies will be required for glucagon, particularly in the context of simultaneous GLP-1R agonism.

Parallel to the development of the glucagon-based co-agonists, investigations from an independent research group validated the concept, reporting anti-obesity properties for a chemically modified version of the gut hormone oxyntomodulin (OXM) [79]. OXM is a peptide hormone released postprandially from the intestinal L cells. It is able to activate both the GcgR and the GLP-1R but with much reduced potency relative to the native ligands. Despite its low potency at both receptors and less-certain in vivo activity at the GcgR, chronic OXM treatment decreased body weight and food intake in rodents when GLP-1R action was present [79–81]. The anorectic and thermogenic effects of OXM were also demonstrated in overweight and obese humans, where chronic administration with native OXM resulted in modest weight loss without any detectable adverse effects [82–84].

Like GLP-1, OXM is rapidly degraded in the circulation by DPP-IV. To improve the pharmacokinetic profile and further explore the therapeutic potential of such an endogenously based GLP-1R/GcgR co-agonist, a DPP-IV-resistant OXM analogue was developed by adding a d-stereoisomer of serine at position 2 and a cholesterol moiety to the C-terminal domain. This modified version of OXM safely improved body weight and lipid metabolism when compared with a long-acting GLP-1R agonist in DIO mice [79]. Interestingly, no evidence of the hyperglycaemic effect of glucagon was detected in the OXM-treated mice. Instead, OXM treatment resulted in improved glycaemic control at a magnitude comparable with that of a selective GLP-1R agonist [79]. Subsequent OXM structure–activity relationship studies identified a series of analogues with superior potency relative to the native hormone [85]. Variants of OXM resistant to DPP-IV (achieved by addition of PEG [86], fatty acids [87], substitution of certain amino acids [85] or even combined modifications [88]) have been advanced to circumvent the limited clinical potential of the native peptide. Such OXM analogues hold promise for the treatment of obesity and independently support the findings with glucagon-based co-agonists.

### Translational perspective for the use of dual GLP-1R/GcgR agonists for obesity

Multiple clinical trials involving GLP-1R/GcgR co-agonists are currently underway (Table 1). Triggered by the first report of successful preclinical tests [17], pharmaceutical companies are strongly committed to the development of this novel therapeutic approach against obesity and type 2 diabetes. Their combined efforts represent a significant number of clinical studies, at different stages and with differing co-agonists (Table 1). Results from these clinical trials are beginning to be disseminated. Recent studies by Sanofi-Aventis and Eli Lilly in phase 1 and 2, respectively, were the first to report and have stimulated further interest in the field. In these first short-term clinical trials, focused on safety and dose optimisation, the administration of two different GLP-1R/GcgR co-agonists reduced HbA_1c_ in overweight diabetic individuals, with some degree of body weight loss [69]. These outcomes are preliminary but suggest that the dual GLP-1R/GcgR agonism observed in preclinical models may translate to humans. They help define the magnitude of GcgR agonism that can be tolerated in the context of GLP-1R agonism, as well as the degree of additional efficacy that it might yet be possible to achieve. Going forward, special attention should be paid to the exploration of potential unwanted effects, particularly those affecting cardiovascular health with heart rate being one of the easiest variables to monitor.

### Co-administration of GLP-1 and glucagon for obesity therapy

Based on the above findings and considering that GLP-1 and glucagon are registered medicines in the treatment of different aspects of diabetes, exploratory combination studies in non-diabetic overweight individuals have been carried out. It was found that acute co-infusion of low doses of GLP-1 and native glucagon reduced food intake and increased energy expenditure to a greater extent than was achieved with either peptide infused alone [89, 90]. There were no reported adverse effects and it was confirmed that GLP-1 prevented the diabetogenic effect of glucagon through its insulinotropic actions. While these results are certainly encouraging, they should be regarded as preliminary. Acute measurements that will require more extensive study are needed before any meaningful conclusion can be made regarding the long-term prospects for combination therapy as mixture or for a single molecule with integrated dual agonism.

## Alternative multi-agonists for obesity treatment

GLP-1R/GcgR dual agonists have shown impressive metabolic benefits and inspired the search for what now represents a growing class of rationally designed multi-agonists with differentiated mechanisms of action for obesity therapy. Other gut hormones, such as glucose-dependent insulinotropic polypeptide (GIP), have been utilised in dual agonists. GIP stimulates insulin secretion in response to nutrient ingestion [14]. Due to sequence similarities between GLP-1 and GIP, a chimera peptide of GLP-1 residues and certain C-terminal residues of GIP activates both the GLP-1 and GIP receptor (GIPR) [91]. A biweekly injection of a fatty-acylated version of this dual agonist improved body weight, glucose homeostasis and lipid metabolism in DIO mice [91]. Chronic treatment with the co-agonist also improved glucose tolerance and insulin secretion in cynomolgus monkeys and humans [91]. Other GLP-1R/GIPR co-agonists such as Cpd86 (Eli Lilly), ZP-DI-70 (Zealand) and NN9709/MAR709 (Novo Nordisk/Marcadia) have also been designed and tested in preclinical models of obesity and subsequently advanced to clinical trials (Table 1).

In addition to dual agonists, triple agonists hold enhanced therapeutic potential for obesity and type 2 diabetes (Table 1). In DIO mice, a GLP-1R/GIPR co-agonist supplemented by an injection of a GcgR agonist resulted in a greater decrease in body weight and food intake than the co-agonist alone [92]. Therefore, a tri-agonist was designed, incorporating residues from GLP-1, glucagon and GIP, as well as having a stabilising CEX. The tri-agonist was tenfold more active than individual native hormones at all three receptors in vitro, and was more effective than a GLP-1R/GIPR co-agonist at lowering body weight in DIO mice [92]. Following this, novel GLP-1R/GIPR/GcgR tri-agonists, achieved through antibody-based constructs, have been developed. The fusion of a co-agonist or a single gut hormone with either the light or heavy chain of the antibody palivizumab provides mixed agonists capable of potently activating two or more receptors simultaneously in a balanced manner [93]. Such an antibody-based fusion protein that activates both the GLP-1R and GcgR is reported to reduce body weight in DIO mice by 12% [93].

While gut-hormone-based multi-agonists have great therapeutic potential, the conceptual approach to multi-agonism is not limited to incretin hormones, or even to structurally related peptides. An intriguing avenue of research is the combination of peptides and nuclear hormones, where the peptide serves to target the latter to specific tissues and intracellular sites of action to minimise adverse off-target tissue effects. For example, it is known that oestrogen is an anorectic hormone that also promotes thermogenesis [94, 95]. Hence, chronic oestrogen therapy may protect against obesity and its related comorbidities. Given alone, oestrogen has oncogenic potential, particularly in gynaecological tissues, precluding its use for metabolic purposes. To minimise unwanted effects and maximise metabolic actions, a covalent conjugate of oestrogen and GLP-1 was developed. This GLP-1-mediated delivery of oestrogen to specific tissues enhanced body weight loss and improved glycaemic control and insulin sensitivity relative to either of the hormones alone in DIO mice [96]. Subsequent studies documented the protective role of the GLP-1/oestrogen conjugate against the loss of pancreatic beta cells in New Zealand Obese (NZO) mice [97]. Importantly, no uterine hypertrophy or tumorigenic effects were detected in mice following chronic intervention with the conjugate, demonstrating its specificity in action and relative safety in comparison with conventional oestrogen treatment.

The initial studies with oestrogen targeted delivery have recently been expanded to include an additional set of matched hormones. A conjugate of glucagon and thyroid hormone (T_3_) has been created to deliver T_3_ specifically to the liver and adipose tissue to improve lipid metabolism, lessen hepatic steatosis and lower body weight. Coordinated action of both hormones synergistically corrected dyslipidaemia and hepatic steatosis in obese and atherosclerotic mice [98]. At the highest doses tested, the combined targeted therapy also decreased body weight. The glucagon-mediated delivery of T_3_ specifically to tissues expressing the GcgR also improved glucose homeostasis. This indicates the ability of T_3_ to override the diabetogenic liability of GcgR agonism, most notably by hepatic action. Importantly, as observed with targeted oestrogen therapy, glucagon delivery of T_3_ demonstrated less of the detrimental cardiovascular effects of thyroid hormone action [98]. These preclinical observations in rodents promote continued study with the aim of translation to larger animals and specifically primates, the central focus being the quantification of therapeutic index when administered for sustained periods. Unlike the combinatorial therapy of G-protein-coupled receptor-based peptide dual agonists, such as GLP-1/glucagon, these peptidyl nuclear hormone conjugates require additional consideration for differential pharmacodynamics where time action for clearance of each activity is inherently dissimilar.

## Perspective and future directions

Development of the first GLP-1R/ GcgR co-agonists has prompted the search for novel multi-agonists of differentiated mechanisms that offer unprecedented potential in the treatment of obesity. Chemical refinement in numerous academic and commercial laboratories continues in the pursuit of optimal pharmaceutical properties. Biologically, the mechanistic underpinnings and subtleties of these novel therapeutics are just beginning to be fully understood, with species- and sex-specific differences remaining high priorities in continuing studies. Integrated preclinical studies combining targeted mouse mutagenesis with pharmacology employing chemically refined agents should accelerate our understanding of biological action. As a specific example: do these multi-agonists act simultaneously at multiple receptors residing at a single tissue or even a single cell? In addition, as with any emerging pharmacology, careful and continued analysis for potential adverse effects is required, especially as these therapies advance for treatments in genetically diverse patient populations. While designed to be more specific in action, there is always the theoretical prospect of reduced safety if an oncogenic precursor happens to express more than one of the responsive receptors. Similarly, immunogenic potential is an ever-present concern in macromolecular medicinal chemistry and dual agonists capable of simultaneously binding at more than one cellular site increase the risk of an immune response [12].

Despite the associated risks, co- and multi-agonists herald the dawn of an era of more personalised metabolic medicine. An expanded portfolio of multi-agonists would allow physicians to tailor therapy that targets specific facets of metabolic disease. For example, GLP-1R/GIPR dual agonists seem preferential in treatment of modestly overweight individuals with diabetes, where the emphasis is on metabolic control with lesser requirement for body weight reduction. In contrast, individuals with dyslipidaemia or hepatic steatosis might benefit more from treatment with a glucagon/T_3_ conjugate where reductions in hepatic fat and circulating lipids are of primary importance. Finally, in obese diabetic individuals, the GLP-1R/GIPR/GcgR tri-agonist may be the most appropriate option, having the greatest potential to improve metabolism by weight-dependent and -independent mechanisms.

Beyond metabolic disease, these multifunctional agonists have the potential to treat diseases where targeted medicinal action is preferred over systematic administration, as demonstrated with the GLP-1/oestrogen conjugate. Similar combinations of active biological agents might minimise the unwanted cardiovascular effects of traditional small-molecule-based drugs or target anti-inflammatory pharmacology to only certain select tissues. In addition, certain diseases such as Allan-Herndon-Dudley syndrome arise from the absence of a single specific transporter. This disease is characterised by the lack of functional MCT8 transporters, which ferry T_3_ into the CNS. The use of conjugates such as GLP-1/T_3_ could serve as a ‘Trojan horse’ delivery system where internalisation through a functional GLP-1 receptor might deliver T_3_ to the brain, ameliorating the disease (and potentially other similar human genetic deficiencies).

The discovery of GLP-1R/GcgR dual agonists has provided a fresh medicinal approach and renewed hope that obesity and its associated abnormalities might be managed with medicines as opposed to surgical interventions. Whether the spectacular preclinical pharmacology of GLP-1R/GcgR co-agonists will translate through successful phase 3 registration trials is a question that will be answered in the coming years, but the pursuit of multifunctional, targeted therapeutics is destined to continue.

## Electronic supplementary material

ESM Downloadable slide (PPTX 145 kb)

## Abbreviations

BAT: Brown adipose tissue
CEX: C-terminal extension
CNS: Central nervous system
DIO: Diet-induced obese
DPP-IV: Dipeptidylpeptidase-IV
EMA: European Medicines Agency
FDA: Food and Drug Administration
GcgR: Glucagon receptor
GIP: Glucose-dependent insulinotropic polypeptide
GIPR: GIP receptor
GLP-1: Glucagon-like peptide 1
GLP-1R: GLP-1 receptor
OXM: Oxyntomodulin
PEG: Polyethylene glycol
STZ: Streptozotocin
T_3_: Thyroid hormone

## References

[1] Obesity and overweight fact sheet. Available from www.who.int/mediacentre/factsheets/fs311/en/. Accessed June 2016

[2] Bray GA (2004). Medical consequences of obesity. J Clin Endocrinol Metab.

[3] Thompson D, Edelsberg J, Colditz GA, Bird AP, Oster G (1999). Lifetime health and economic consequences of obesity. Arch Intern Med.

[4] Roglic G, World Health Organization (2016). Global report on diabetes.

[5] Guariguata L, Whiting DR, Hambleton I, Beagley J, Linnenkamp U, Shaw JE (2014). Global estimates of diabetes prevalence for 2013 and projections for 2035. Diabetes Res Clin Pract.

[6] Kraschnewski JL, Boan J, Esposito J (2010). Long-term weight loss maintenance in the United States. Int J Obes.

[7] Maggard-Gibbons M, Maglione M, Livhits M (2013). Bariatric surgery for weight loss and glycemic control in nonmorbidly obese adults with diabetes: a systematic review. JAMA.

[8] Chang SH, Stoll CR, Song J, Varela JE, Eagon CJ, Colditz GA (2014). The effectiveness and risks of bariatric surgery: an updated systematic review and meta-analysis, 2003-2012. JAMA Surg.

[9] (1992) Gastrointestinal surgery for severe obesity: National Institutes of Health Consensus Development Conference Statement March 25–27 1991. Am J Clin Nutr 55: 615S–619S 10.1093/ajcn/55.2.615s 1733140

[10] Apovian CM, Garvey WT, Ryan DH (2015). Challenging obesity: patient, provider, and expert perspectives on the roles of available and emerging nonsurgical therapies. Obesity.

[11] Magkos F, Fraterrigo G, Yoshino J (2016). Effects of moderate and subsequent progressive weight loss on metabolic function and adipose tissue biology in humans with obesity. Cell Metab.

[12] Apovian CM, Aronne LJ, Bessesen DH (2015). Pharmacological management of obesity: an Endocrine Society clinical practice guideline. J Clin Endocrinol Metab.

[13] Wellman PJ, Maher TJ (1999). Synergistic interactions between fenfluramine and phentermine. Int J Obes Relat Metab Disord.

[14] Baggio LL, Drucker DJ (2007). Biology of incretins: GLP-1 and GIP. Gastroenterology.

[15] Todd JF, Wilding JP, Edwards CM, Khan FA, Ghatei MA, Bloom SR (1997). Glucagon-like peptide-1 (GLP-1): a trial of treatment in non-insulin-dependent diabetes mellitus. Eur J Clin Investig.

[16] von Websky K, Reichetzeder C, Hocher B (2014). Physiology and pathophysiology of incretins in the kidney. Curr Opin Nephrol Hypertens.

[17] Day JW, Ottaway N, Patterson JT (2009). A new glucagon and GLP-1 co-agonist eliminates obesity in rodents. Nat Chem Biol.

[18] Finan B, Clemmensen C, Muller TD (2015). Emerging opportunities for the treatment of metabolic diseases: glucagon-like peptide-1 based multi-agonists. Mol Cell Endocrinol.

[19] Lorenz M, Evers A, Wagner M (2013). Recent progress and future options in the development of GLP-1 receptor agonists for the treatment of diabesity. Bioorg Med Chem Lett.

[20] Cornell S (2012). Differentiating among incretin therapies: a multiple-target approach to type 2 diabetes. J Clin Pharm Ther.

[21] Painter NA, Morello CM, Singh RF, McBane SE (2013). An evidence-based and practical approach to using Bydureon™ in patients with type 2 diabetes. J Am Board Fam Med.

[22] Meier JJ (2012). GLP-1 receptor agonists for individualized treatment of type 2 diabetes mellitus. Nat Rev Endocrinol.

[23] Rosenstock J, Raccah D, Koranyi L (2013). Efficacy and safety of lixisenatide once daily versus exenatide twice daily in type 2 diabetes inadequately controlled on metformin: a 24-week, randomized, open-label, active-controlled study (GetGoal-X). Diabetes Care.

[24] Fonseca VA, Alvarado-Ruiz R, Raccah D (2012). Efficacy and safety of the once-daily GLP-1 receptor agonist lixisenatide in monotherapy: a randomized, double-blind, placebo-controlled trial in patients with type 2 diabetes (GetGoal-Mono). Diabetes Care.

[25] Russell-Jones D, Cuddihy RM, Hanefeld M (2012). Efficacy and safety of exenatide once weekly versus metformin, pioglitazone, and sitagliptin used as monotherapy in drug-naive patients with type 2 diabetes (DURATION-4): a 26-week double-blind study. Diabetes Care.

[26] Knudsen LB, Nielsen PF, Huusfeldt PO (2000). Potent derivatives of glucagon-like peptide-1 with pharmacokinetic properties suitable for once daily administration. J Med Chem.

[27] Ostawal A, Mocevic E, Kragh N, Xu W (2016). Clinical effectiveness of liraglutide in type 2 diabetes treatment in the real-world setting: a systematic literature review. Diabetes Ther.

[28] Lau J, Bloch P, Schaffer L (2015). Discovery of the once-weekly glucagon-like peptide-1 (GLP-1) analogue semaglutide. J Med Chem.

[29] Gotfredsen CF, Molck AM, Thorup I (2014). The human GLP-1 analogs liraglutide and semaglutide: absence of histopathological effects on the pancreas in nonhuman primates. Diabetes.

[30] Nauck MA, Petrie JR, Sesti G (2016). A phase 2, randomized, dose-finding study of the novel once-weekly human GLP-1 analog, semaglutide, compared with placebo and open-label liraglutide in patients with type 2 diabetes. Diabetes Care.

[31] Marso SP, Bain SC, Consoli A (2016). Semaglutide and cardiovascular outcomes in patients with type 2 diabetes. N Engl J Med.

[32] Thompson AM, Trujillo JM (2015). Dulaglutide: the newest GLP-1 receptor agonist for the management of type 2 diabetes. Ann Pharmacother.

[33] Jendle J, Grunberger G, Blevins T, Giorgino F, Hietpas RT, Botros FT (2016). Efficacy and safety of dulaglutide in the treatment of type 2 diabetes: a comprehensive review of the dulaglutide clinical data focusing on the AWARD phase 3 clinical trial program. Diabetes Metab Res Rev.

[34] Rosenstock J, Reusch J, Bush M (2009). Potential of albiglutide, a long-acting GLP-1 receptor agonist, in type 2 diabetes: a randomized controlled trial exploring weekly, biweekly, and monthly dosing. Diabetes Care.

[35] Troke RC, Tan TM, Bloom SR (2014). The future role of gut hormones in the treatment of obesity. Ther Adv Chronic Dis.

[36] Elashoff M, Matveyenko AV, Gier B, Elashoff R, Butler PC (2011). Pancreatitis, pancreatic, and thyroid cancer with glucagon-like peptide-1-based therapies. Gastroenterology.

[37] Gier B, Matveyenko AV, Kirakossian D, Dawson D, Dry SM, Butler PC (2012). Chronic GLP-1 receptor activation by exendin-4 induces expansion of pancreatic duct glands in rats and accelerates formation of dysplastic lesions and chronic pancreatitis in the Kras(G12D) mouse model. Diabetes.

[38] Nachnani JS, Bulchandani DG, Nookala A (2010). Biochemical and histological effects of exendin-4 (exenatide) on the rat pancreas. Diabetologia.

[39] Nyborg NC, Molck AM, Madsen LW, Knudsen LB (2012). The human GLP-1 analog liraglutide and the pancreas: evidence for the absence of structural pancreatic changes in three species. Diabetes.

[40] Pfeffer MA, Claggett B, Diaz R (2015). Lixisenatide in patients with type 2 diabetes and acute coronary syndrome. N Engl J Med.

[41] Marso SP, Daniels GH, Brown-Frandsen K (2016). Liraglutide and cardiovascular outcomes in type 2 diabetes. N Engl J Med.

[42] Drucker DJ (2005). Biologic actions and therapeutic potential of the proglucagon-derived peptides. Nat Clin Pract Endocrinol Metab.

[43] Lee YH, Wang MY, Yu XX, Unger RH (2016). Glucagon is the key factor in the development of diabetes. Diabetologia.

[44] Liang Y, Osborne MC, Monia BP (2004). Reduction in glucagon receptor expression by an antisense oligonucleotide ameliorates diabetic syndrome in *db*/*db* mice. Diabetes.

[45] Cryer PE (2012). Minireview: glucagon in the pathogenesis of hypoglycemia and hyperglycemia in diabetes. Endocrinology.

[46] Wild D, von Maltzahn R, Brohan E, Christensen T, Clauson P, Gonder-Frederick L (2007). A critical review of the literature on fear of hypoglycemia in diabetes: implications for diabetes management and patient education. Patient Educ Couns.

[47] Chabenne JR, DiMarchi MA, Gelfanov VM, DiMarchi RD (2010). Optimization of the native glucagon sequence for medicinal purposes. J Diabetes Sci Technol.

[48] Gelling RW, Du XQ, Dichmann DS (2003). Lower blood glucose, hyperglucagonemia, and pancreatic alpha cell hyperplasia in glucagon receptor knockout mice. Proc Natl Acad Sci U S A.

[49] Lee Y, Wang MY, Du XQ, Charron MJ, Unger RH (2011). Glucagon receptor knockout prevents insulin-deficient type 1 diabetes in mice. Diabetes.

[50] Qureshi SA, Rios Candelore M, Xie D (2004). A novel glucagon receptor antagonist inhibits glucagon-mediated biological effects. Diabetes.

[51] Sloop KW, Cao JX, Siesky AM (2004). Hepatic and glucagon-like peptide-1-mediated reversal of diabetes by glucagon receptor antisense oligonucleotide inhibitors. J Clin Invest.

[52] Johnson DG, Goebel CU, Hruby VJ, Bregman MD, Trivedi D (1982). Hyperglycemia of diabetic rats decreased by a glucagon receptor antagonist. Science.

[53] Petersen KF, Sullivan JT (2001). Effects of a novel glucagon receptor antagonist (Bay 27-9955) on glucagon-stimulated glucose production in humans. Diabetologia.

[54] Kazda CM, Ding Y, Kelly RP (2016). Response to comment on Kazda et al. evaluation of efficacy and safety of the glucagon receptor antagonist LY2409021 in patients with type 2 diabetes: 12- and 24-week phase 2 studies. Diabetes Care 2016;39:1241-1249. Diabetes Care.

[55] Kelly RP, Garhyan P, Raddad E (2015). Short-term administration of the glucagon receptor antagonist LY2409021 lowers blood glucose in healthy people and in those with type 2 diabetes. Diabetes Obes Metab.

[56] Habegger KM, Heppner KM, Geary N, Bartness TJ, DiMarchi R, Tschop MH (2010). The metabolic actions of glucagon revisited. Nat Rev Endocrinol.

[57] Perea A, Clemente F, Martinell J, Villanueva-Penacarrillo ML, Valverde I (1995). Physiological effect of glucagon in human isolated adipocytes. Horm Metab Res.

[58] Slavin BG, Ong JM, Kern PA (1994). Hormonal regulation of hormone-sensitive lipase activity and mRNA levels in isolated rat adipocytes. J Lipid Res.

[59] Davidson IWF, Salter JM, Best CH (1960). The effect of glucagon on the metabolic rate of rats. Am J Clin Nutr.

[60] Nair KS (1987). Hyperglucagonemia increases resting metabolic rate in man during insulin deficiency. J Clin Endocrinol Metab.

[61] Joel CD (1966). Stimulation of metabolism of rat brown adipose tissue by addition of lipolytic hormones in vitro. J Biol Chem.

[62] Kuroshima A, Yahata T (1979). Thermogenic responses of brown adipocytes to noradrenaline and glucagon in heat-acclimated and cold-acclimated rats. Jpn J Physiol.

[63] Doi K, Kuroshima A (1982). Modified metabolic responsiveness to glucagon in cold-acclimated and heat-acclimated rats. Life Sci.

[64] Salem V, Izzi-Engbeaya C, Coello C (2016). Glucagon increases energy expenditure independently of brown adipose tissue activation in humans. Diabetes Obes Metab.

[65] Miyoshi H, Shulman GI, Peters EJ, Wolfe MH, Elahi D, Wolfe RR (1988). Hormonal control of substrate cycling in humans. J Clin Invest.

[66] Sammons MF, Lee EC (2015). Recent progress in the development of small-molecule glucagon receptor antagonists. Bioorg Med Chem Lett.

[67] Kazda CM, Ding Y, Kelly RP (2016). Evaluation of efficacy and safety of the glucagon receptor antagonist LY2409021 in patients with type 2 diabetes: 12- and 24-week phase 2 studies. Diabetes Care.

[68] Kazierad DJ, Bergman A, Tan B (2016). Effects of multiple ascending doses of the glucagon receptor antagonist PF-06291874 in patients with type 2 diabetes mellitus. Diabetes Obes Metab.

[69] Tschop MH, Finan B, Clemmensen C (2016). Unimolecular polypharmacy for treatment of diabetes and obesity. Cell Metab.

[70] Heppner KM, Perez-Tilve D (2015). GLP-1 based therapeutics: simultaneously combating T2DM and obesity. Front Neurosci.

[71] Zhang Y, Proenca R, Maffei M, Barone M, Leopold L, Friedman JM (1994). Positional cloning of the mouse obese gene and its human homologue. Nature.

[72] Heymsfield SB, Greenberg AS, Fujioka K (1999). Recombinant leptin for weight loss in obese and lean adults: a randomized, controlled, dose-escalation trial. JAMA.

[73] Clemmensen C, Chabenne J, Finan B (2014). GLP-1/glucagon coagonism restores leptin responsiveness in obese mice chronically maintained on an obesogenic diet. Diabetes.

[74] Sandoval DA, D'Alessio DA (2015). Physiology of proglucagon peptides: role of glucagon and GLP-1 in health and disease. Physiol Rev.

[75] Sisley S, Gutierrez-Aguilar R, Scott M, D'Alessio DA, Sandoval DA, Seeley RJ (2014). Neuronal GLP1R mediates liraglutide’s anorectic but not glucose-lowering effect. J Clin Invest.

[76] Abraham MA, Lam TK (2016). Glucagon action in the brain. Diabetologia.

[77] Lao J, Hansen BC, Dimarchi R, Pocai A (2013). Effect of GLP1R/GCGR dual agonist in monkeys. Diabetes.

[78] Henderson SJ, Konkar A, Hornigold DC (2016). Robust anti-obesity and metabolic effects of a dual GLP-1/glucagon receptor peptide agonist in rodents and non-human primates. Diabetes Obes Metab.

[79] Pocai A, Carrington PE, Adams JR (2009). Glucagon-like peptide 1/glucagon receptor dual agonism reverses obesity in mice. Diabetes.

[80] Dakin CL, Gunn I, Small CJ (2001). Oxyntomodulin inhibits food intake in the rat. Endocrinology.

[81] Dakin CL, Small CJ, Park AJ, Seth A, Ghatei MA, Bloom SR (2002). Repeated ICV administration of oxyntomodulin causes a greater reduction in body weight gain than in pair-fed rats. Am J Physiol Endocrinol Metab.

[82] Wynne K, Park AJ, Small CJ (2005). Subcutaneous oxyntomodulin reduces body weight in overweight and obese subjects: a double-blind, randomized, controlled trial. Diabetes.

[83] Wynne K, Park AJ, Small CJ (2006). Oxyntomodulin increases energy expenditure in addition to decreasing energy intake in overweight and obese humans: a randomised controlled trial. Int J Obes.

[84] Cohen MA, Ellis SM, Le Roux CW (2003). Oxyntomodulin suppresses appetite and reduces food intake in humans. J Clin Endocrinol Metab.

[85] Santoprete A, Capito E, Carrington PE (2011). DPP-IV-resistant, long-acting oxyntomodulin derivatives. J Pept Sci.

[86] Bianchi E, Carrington PE, Ingallinella P (2013). A PEGylated analog of the gut hormone oxyntomodulin with long-lasting antihyperglycemic, insulinotropic and anorexigenic activity. Bioorg Med Chem.

[87] Druce MR, Minnion JS, Field BC (2009). Investigation of structure-activity relationships of Oxyntomodulin (Oxm) using Oxm analogs. Endocrinology.

[88] Kerr BD, Flatt PR, Gault VA (2010). (D-nn2)Oxm[mPEG-PAL]: a novel chemically modified analogue of oxyntomodulin with antihyperglycaemic, insulinotropic and anorexigenic actions. Biochem Pharmacol.

[89] Tan TM, Field BC, McCullough KA (2013). Coadministration of glucagon-like peptide-1 during glucagon infusion in humans results in increased energy expenditure and amelioration of hyperglycemia. Diabetes.

[90] Cegla J, Troke RC, Jones B (2014). Coinfusion of low-dose GLP-1 and glucagon in man results in a reduction in food intake. Diabetes.

[91] Finan B, Ma T, Ottaway N (2013). Unimolecular dual incretins maximize metabolic benefits in rodents, monkeys, and humans. Sci Transl Med.

[92] Finan B, Yang B, Ottaway N (2015). A rationally designed monomeric peptide triagonist corrects obesity and diabetes in rodents. Nat Med.

[93] Wang Y, Du J, Zou H (2016). Multifunctional antibody agonists targeting glucagon-like peptide-1, glucagon, and glucose-dependent insulinotropic polypeptide receptors. Angew Chem Int Ed Eng.

[94] Mauvais-Jarvis F (2011). Estrogen and androgen receptors: regulators of fuel homeostasis and emerging targets for diabetes and obesity. Trends Endocrinol Metab.

[95] Tiano JP, Delghingaro-Augusto V, Le May C (2011). Estrogen receptor activation reduces lipid synthesis in pancreatic islets and prevents beta cell failure in rodent models of type 2 diabetes. J Clin Invest.

[96] Finan B, Yang B, Ottaway N (2012). Targeted estrogen delivery reverses the metabolic syndrome. Nat Med.

[97] Schwenk RW, Baumeier C, Finan B (2015). GLP-1-oestrogen attenuates hyperphagia and protects from beta cell failure in diabetes-prone New Zealand obese (NZO) mice. Diabetologia.

[98] Finan B, Clemmensen C, Zhu Z (2016). Chemical hybridization of glucagon and thyroid hormone optimizes therapeutic impact for metabolic disease. Cell.

