# Supplementary material for: GLP-1/glucagon receptor co-agonism for treatment of obesity
Source: Diabetologia. 2017 Jul 21;60(10):1851–61. doi: 10.1007/s00125-017-4354-8 (PMC6448809; doi:10.1007/s00125-017-4354-8)
Supplement: Supplementary file 1 — (PPTX 145 kb) [file 125_2017_4354_MOESM1_ESM.pptx]

## Slide 1
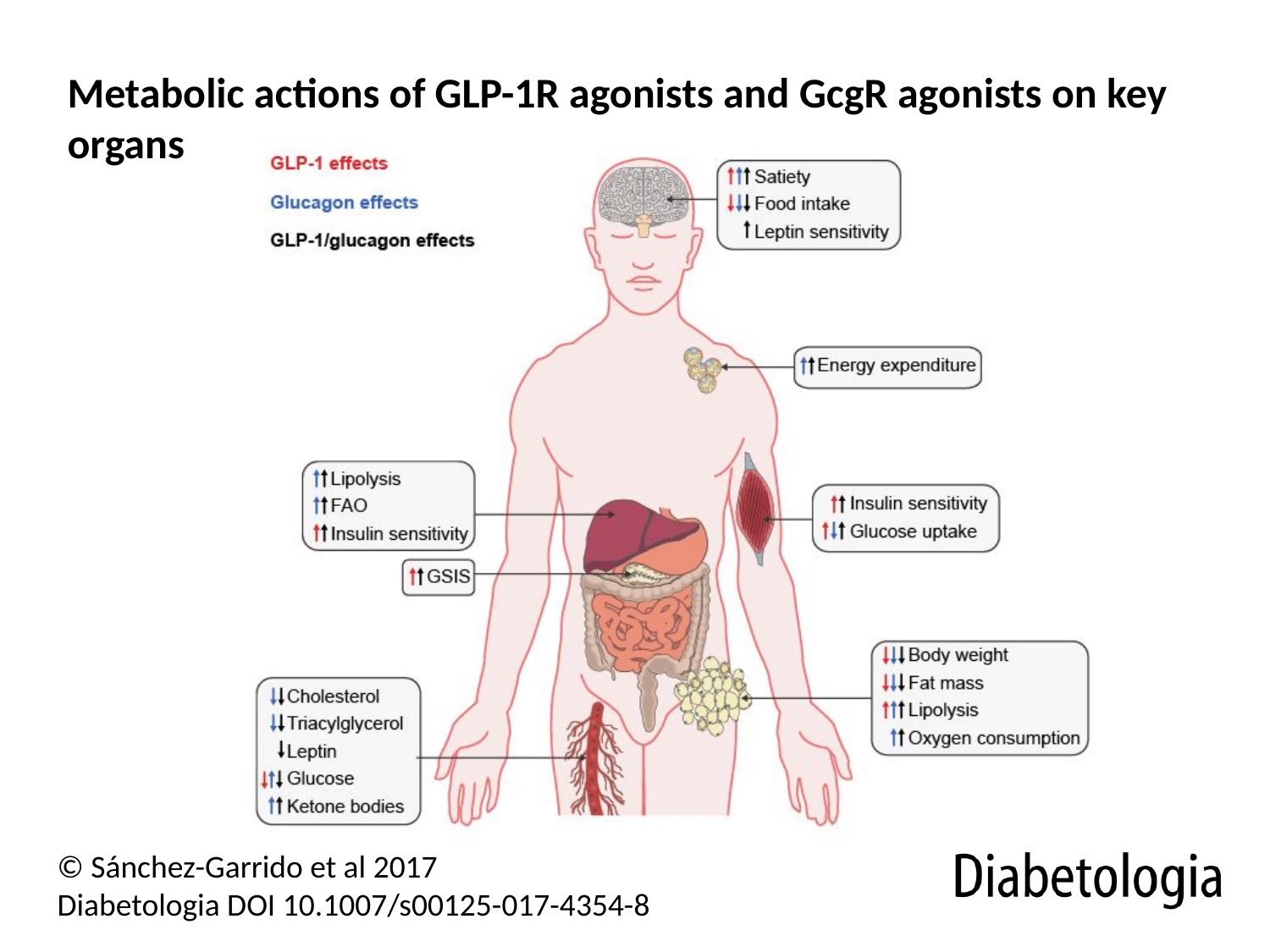

Metabolic actions of GLP-1R agonists and GcgR agonists on key organs
# Insert figure
© Sánchez-Garrido et al 2017
Diabetologia DOI 10.1007/s00125-017-4354-8
